# Mortality Statistics

**Published:** 1908-07

**Authors:** 


### MORTALITY STATISTICS.

The reports of the Census office has recently been issued for the year 1906. Unfortunately only 15 of the 46 states are accepted as registration states, but the Bureau of the census is making earnest effort to increase this number in order that the United States may obtain a complete and uniform system of registration. The data upon which these reports are based are not collected by the National Government, but taken from the registration under state laws and municipal ordinances. Naturally it is exceedingly difficult to secure uniform and comparable data from so many different sources and a very important part of the function of the national registration office is to promote the use of identical methods and to urge the enforcement of the city and state laws.

There were 658,105 deaths reported during the year 1906 from the registration area which has an estimated population of 40,996,317 persons, and the death rate was consequently 16.1 per 1,000. The death rate of the colored population is much higher than that of the white population. For the five-year period, 1901 to 1905, the death rate of the whites was 17.5 and that of the colored population was 28.4 per 1,000 or over 60 per cent. greater. About the same relation is shown for the year 1906. If the death rate of the colored population could be further sub-divided it would undoubtedly be found that the mulatto shows a higher death rate than the black negro. This fact alone should be sufficient to make those who claim to favor miscegenation hesitate in advancing their repulsive scheme.

Diabetes is the only disease which has shown a steadily increasing rate for the six years given in the tables. An interesting point in this connection is the claim of Eccles* that with an increase in the wealth of a nation comes an increase in the number of cases of diabetes, and *pari passu* with this, an increase in the consumption of proteid, with a corresponding decrease in the consumption of carbohydrates.

There were 13,160 deaths from typhoid fever reported from the registration area during the year 1906. Only 95 deaths were reported from small pox, while 5,087 were caused by measles.

*Medical Record, New York, May 9, 1908.

Cancer is given as the cause of 29,020. The number of deaths from violence was 49,552. Tuberculosis in all its forms caused 75,512 deaths, and the death rate of 184.2 per thousand is considerably lower than that of any other year shown. Over 86 per cent. of all deaths from tuberculosos are assigned to the pulmonary form.

